# A critical time window for recovery extends beyond one-year post-stroke

**DOI:** 10.1152/jn.00762.2018

**Published:** 2019-05-29

**Authors:** Belén Rubio Ballester, Martina Maier, Armin Duff, Mónica Cameirão, Sergi Bermúdez, Esther Duarte, Ampar Cuxart, Susana Rodríguez, Rosa María San Segundo Mozo, Paul F. M. J. Verschure

**Affiliations:** ^1^Laboratory of Synthetic Perceptive, Emotive and Cognitive Systems, Institute for Bioengineering of Catalonia (IBEC), Barcelona, Spain; ^2^Madeira Interactive Technologies Institute and Universidade da Madeira, Campus Universitário da Penteada, Funchal, Portugal; ^3^Servei de Medicina Física i Rehabilitació, Hospitals del Mar i l’Esperança, Institut Hospital del Mar d’Investigacions Mèdiques, Barcelona, Spain; ^4^Servei de Medicina Física i Rehabilitació, Hospital Universitari Vall d’Hebron, Barcelona, Spain; ^5^Servei de Medicina Física i Rehabilitació, Hospital Joan XIII, Tarragona, Spain; ^6^ICREA, Institució Catalana de Recerca i Estudis Avançats, Passeig Lluís Companys, Barcelona, Spain

**Keywords:** motor recovery, neuroplasticity, neurorehabilitation, stroke recovery, virtual reality

## Abstract

The impact of rehabilitation on post-stroke motor recovery and its dependency on the patient’s chronicity remain unclear. The field has widely accepted the notion of a proportional recovery rule with a “critical window for recovery” within the first 3–6 mo poststroke. This hypothesis justifies the general cessation of physical therapy at chronic stages. However, the limits of this critical window have, so far, been poorly defined. In this analysis, we address this question, and we further explore the temporal structure of motor recovery using individual patient data from a homogeneous sample of 219 individuals with mild to moderate upper-limb hemiparesis. We observed that improvement in body function and structure was possible even at late chronic stages. A bootstrapping analysis revealed a gradient of enhanced sensitivity to treatment that extended beyond 12 mo poststroke. Clinical guidelines for rehabilitation should be revised in the context of this temporal structure.

**NEW & NOTEWORTHY** Previous studies in humans suggest that there is a 3- to 6-mo “critical window” of heightened neuroplasticity poststroke. We analyze the temporal structure of recovery in patients with hemiparesis and uncover a precise gradient of enhanced sensitivity to treatment that expands far beyond the limits of the so-called critical window. These findings highlight the need for providing therapy to patients at the chronic and late chronic stages.

## INTRODUCTION

The absolute incidence of stroke will continue to rise globally with a predicted 12 million stroke deaths in 2030 and 60 million stroke survivors worldwide (Eilers 2003). Stroke leads to focal lesions in the brain due to cell death following hypoxia and inflammation, affecting both gray and white matter tracts (Corbetta et al. 2015). After a stroke, a wide range of deficits can occur with varying onset latencies such as hemiparesis, abnormal posture, spatial hemineglect, aphasia, and spasticity, along with affective and cognitive deficits, chronic pain, and depression (Teasell et al. 2003). Due to improved treatment procedures during the acute stage of stroke (e.g., thrombolysis and thrombectomy), the associated reduction in stroke mortality has led to a greater proportion of patients facing impairments and needing long-term care and rehabilitation. However, prevention, diagnostics, rehabilitation, and prognostics of stroke recovery have not kept pace (Veerbeek et al. 2014).

Motor recovery after stroke has been widely operationalized as the individual’s change in two domains: *1*) body function and structure (WHO 2001), whose improvement has been called “true recovery” (Bernhardt et al. 2017) and refers to the restitution of a movement repertoire that the individual had before the injury; and *2*) the ability to successfully perform the activities of daily living (Levin et al. 2009). While the former is mainly due to the interaction of poststroke plasticity mechanisms and sensorimotor training, the latter is also influenced by the use of explicit and implicit compensatory strategies (Bernhardt et al. 2017; Kwakkel et al. 2017). The most accepted measure for recovery of body function and structure is the change in the Fugl-Meyer Assessment of the upper extremity (UE-FM) scores (Kwakkel et al. 2017), while other clinical scales focus on the assessment of activities, such as the Chedoke Arm and Hand Activity Inventory (CAHAI) (Barreca et al. 2005) or the Barthel Index for Activities of Daily Living (BI) (Granger et al. 1979).

Poststroke motor recovery mostly follows a nonlinear trajectory that reaches asymptotic levels a few months after the injury (Kwakkel et al. 2004). This model suggests the existence of a period of heightened plasticity in which the patient seems to be more responsive to treatment, the so-called “critical window” for recovery. Aiming at characterizing the temporal structure of recovery, animal models and clinical research have identified a combination of mechanisms underlying neurological repair that seems to be unique to the injured brain, including neurogenesis, gliogenesis, axonal sprouting, and the rebalancing of excitation and inhibition in cortical networks (Ward 2017). This state of enhanced plasticity seems to be transient and interacts closely with sensorimotor training to facilitate the recovery of motor function (Zeiler et al. 2016). However, there is no clear evidence of the exact temporal structure of enhanced responsiveness to treatment in humans, and as a result the optimal timing and intensity of treatment remain unclear. A systematic review of 14 studies suggested that, on average, recovery reaches a plateau at 15 wk poststroke for patients with severe hemiparesis and at 6.5 wk for patients with mild hemiparesis (Hendricks et al. 2002). This study however failed to conduct a meta-analysis due to substantial heterogeneity of the sample and protocols. Currently, an ongoing clinical trial is investigating the existence and the duration of a critical window of enhanced neuroplasticity in humans following ischemic stroke (McDonnell et al. 2015). Based on the assumption of the existence of this critical period, the SMARTS 2 trial (NCT02292251) (Krakauer and Cortés 2018) is currently investigating the effect of early and intensive therapy on upper extremity motor recovery. Sharing the same research question, the Critical Periods After Stroke Study (CPASS) is a large ongoing randomized controlled trial that focuses on determining the optimal time after stroke for intensive motor training (Dromerick et al. 2015). To contribute to the delineation of a temporal structure of stroke recovery in humans, we performed an analysis of individual patient clinical data from 219 subjects with upper-limb hemiparesis, who followed occupational therapy (OT) or a virtual reality (VR)-based training protocol using the Rehabilitation Gaming System (RGS) (Cameirão et al. 2010) (Fig. S1 in Supplemental Material; all Supplemental material is available at https://doi.org/10.5281/zenodo.3246368). We show that physical therapy has a significant impact on the function of the upper extremity (UE) at all periods poststroke considered, uncovering a gradient of responsiveness to treatment that extends >12 mo poststroke.

## MATERIALS AND METHODS

### 

#### Data sets.

In this analysis, we included individual patient data from a set of protocols for the recovery of upper extremity function. These protocols included interventions combining OT and a specific VR training protocol (RGS) (Section 1 in Supplemental Material). Participants met the following inclusion criteria: *1*) ischemic strokes (middle cerebral artery territory) or hemorrhagic strokes (intracerebral); *2*) mild-to-moderate upper limb hemiparesis (Medical Research Council scale for proximal muscles >2) after a first-ever stroke; *3*) age between 45 and 85 yr old; and *4*) the absence of any significant cognitive impairment (Mini-Mental State Evaluation >22). All research on human subjects reported in this manuscript was prospectively approved by the Institutional Review Board of Hospital Joan XXIII, Hospital Vall d’Hebron and Hospital del Mar i l’Esperança from Catalonia, and all participants provided written informed consent.

The data sets were divided into 17 conditions depending on the specific characteristics of the patients and the requirements of the treatment provided (Table 1). Most of the RGS conditions included in this analysis have an identical design based on the same set of neurorehabilitation principles (Maier et al. 2019) (see Supplemental Material for a full description of the system and its mechanisms). However, two relevant design differences should be noticed. First, in two protocols used with acute patients the dosage was three days a week instead of five (*conditions 5* and *6* in Table 1). Second, in one study the implicit feedback is augmented through haptic actuators (*condition 14* in Table 1, RGS Haptics).

**Table 1.** Overview of therapy conditions

ID	Group	Average Chronicity	Intervention	*N*	Mean Age (SD)	TSO (SD)	HA (%left)	Sex (%men)	Oxf. Class.	References
*Acute stage*										
1	Control	Acute	12w;5d/w;20min	5	69 (19)	9 (15)	40	80	2/1/0/1/1	(Duff et al. 2013)
2	RGS	Acute	3w;5d/w;20min	5	70 (22)	11 (4)	60	20	1/2/0/1/1	(Duff et al. 2013; Rodriguez et al. 2011)
3	RGS	Acute	12w;3d/w;20min	10	63.5 (29)	11 (17)	40	30	2/2/2/3/1	(da Silva Cameirão et al. 2011; Duff et al. 2013)
4	Control	Acute	3w;5d/w;20min	5	64 (16)	13 (5)	60	60	2/2/0/0/1	(Duff et al. 2013; Rodriguez et al. 2011)
5	Control	Acute	12w;3d/w;20min; NSG	4	65 (28)	13 (12)	75	50	1/0/1/1/1	(da Silva Cameirão et al. 2011)
6	Control	Acute	12w;3d/w;20min; IOT	5	56 (27)	15 (11)	40	40	1/1/2/0/1	(da Silva Cameirão et al. 2011)
*Subacute stage*										
7	RGS	Subacute	3w;5d/w;20min	49	61 (43)	70 (375)	30.6	30.6	11/9/13/1/15	(See Supplemental Material)
8	Control	Subacute	3w;5d/w;20min	4	57 (17)	90 (226)	0	50	4/0/0/0/0	(See Supplemental Material)
*Early chronic stage*										
9	RGS	Chronic	6w;5d/w;30min; +AM	9	63 (31)	400 (5,805)	33.3	33.3		(Ballester et al. 2016)
10	RGS	Chronic	6w;5d/w;30min	9	57 (36)	735 (4,471)	11.1	55.6		(Ballester et al. 2016)
11	Control	Chronic	3w;5d/w;20min; domiciliary	18	68.5 (40)	751 (1,536)	33.3	50	6/2/4/0/6	(Ballester et al. 2017; Nirme et al. 2013)
12	RGS	Chronic	3w;5d/w;20min; +OT	20	64.5 (37)	770 (2,789)	40	30	7/0/4/0/9	(Duff et al. 2013)
*Late chronic stage*										
13	RGS	Late Chronic	3w;5d/w;20min; domiciliary	17	61.5 (43)	997 (2,987)	47.1	35.3	4/3/4/0/6	(Ballester et al. 2017; Nirme et al. 2013)
14	RGS	Late Chronic	4w;5d/w;30min; haptics	14	63 (45)	1,051 (3,250)	50	57.1	6/0/4/1/3	(Cameirão et al. 2012)
15	RGS	Late Chronic	3w;5d/w;30min	15	58 (60)	1,261 (2,041)	33.3	46.7	0/1/2/0/12	(Duff et al. 2013)
16	RGS	Late Chronic	4w;5d/w;30min	16	69.5 (46)	1,536 (3,891)	43.8	62.5	6/4/5/0/1	(Cameirão et al. 2012)
17	RGS	Late Chronic	4w;5d/w;30min; exoskeleton	14	60 (32)	1,758 (2,880)	35.7	71.4	3/1/4/1/5	(Cameirão et al. 2012)

Intervention: duration of included protocols indicated per number of weeks (w), days per week (d/w), and minutes (min) of occupational therapy (OT) and virtual reality (VR)-based therapy per day. AM, condition including the amplification of movements in VR; HA, percentage of patients with left hemisphere affected; Haptics, condition including delivery of haptic feedback during training; IOT, condition including intensive occupational therapy; *N*, sample size in the experimental group; NSG, protocol based on nonspecific gaming system (i.e., Nintendo Wii); Oxf. Class., count of stroke types [lacunar stroke (LACS)/partial anterior circulation stroke (PACS)/total anterior circulation stroke (TACS)/or posterior circulation stroke (POCS)] according to the Oxford Stroke Classification scale; RGS, Rehabilitation Gaming System; Sex, percentage of men; TSO, median (maximum − minimum) days since the stroke.

Furthermore, all RGS protocols used by acute and subacute patients combined RGS-based training with supervised OT, while *condition 13* tested the application of RGS in a domiciliary setting. Despite these differences, in all RGS conditions the same protocol was used. A formal risk of bias analysis on the primary outcome (i.e., change in the UE-FM) was performed using ROBINS-I tool (Table S1 in Supplemental Material) covering the evaluation of confounding variables, recruitment for participants, intervention classification, deviations from intended interventions, missing data, measurement of outcomes, and selection of reported results.

#### Outcome measures.

The primary outcome considered in this analysis was the UE motor impairment and activity at the end of therapy, as measured by two standardized clinical scales: the UE-FM and CAHAI (Barreca et al. 2005) scales. Previous studies have shown that the UE-FM shows excellent reliability, responsiveness, and validity properties (Wei et al. 2011). Second, the CAHAI evaluates the UE bilateral function in the performance of specific iADLs (Barreca et al. 2005). Score changes in the UE-FM and CAHAI were used as measures of motor improvement (body function and structure) and performance in Instrumental Activities of Daily Living (iADLs), respectively. In addition, the BI (Granger et al. 1979) was considered a secondary outcome for the assessment of the patient’s level of independence.

#### Statistical analysis.

We performed two analyses. First, we explored recovery measures independently within each of the 17 conditions. In this analysis, we examined recovery measures using the UE-FM and CAHAI in absolute terms. Here, we quantified improvement using mean differences and 95% confidence intervals (CI). Second, we analyzed the temporal structure of recovery poststroke by merging all the conditions (178 patients performing RGS-based training and 368 follow-up measures) and bootstrapping our data using the Efron and Tib method (Efron and Tibshirani 1986) to evaluate the effects of the therapy across the patients’ chronicity (subacute, early chronic, and late chronic). This method overcomes the high intersubject variability and provides a superior statistical power (Duff et al. 2011). The homogenized data were generated by separating improvement measures at different time intervals and allocating them to either being an RGS-based training, OT based-training, or follow-up (i.e., no therapy). We then calculated the improvement rate per week normalized within subjects according to their respective recovery potential. This metric therefore captures the improvement observed normalized to the total amount that each patient can gain given the baseline of each in standardized clinical scales. The normalized improvement (NI) on scale *i* at time *t* was defined as:

(1) $$NI\left(i,t\right)=\left[\frac{X_{i}\left(t\right)-X_{i}\left(t=0\right)}{MAxScore_{i}-X_{i}\left(t=0\right)}\right]\times 100$$

where *X_i_* (*t* = 0) refers to the corresponding baseline score. According to this normalization method, a patient with a baseline score of 16 in UE-FM will have 50 points of potential improvement since the UE-FM has a maximum score of 66 (da Silva Cameirão et al. 2011). In case this patient would recover 10 points, reaching a score of 26 points in the scale at T1, this would be equal to 20% (i.e., 10/50) of NI. Note that this value may depend on the time lapsed between baseline measurement and subsequent assessments. We overcome this bias by dividing the NI by the number of weeks between both measurement time points, therefore obtaining an estimate of NI per week. This normalization method allowed us to bundle the data of the different conditions while overcoming the risk of bias due to the variation in treatment intensity and response rates among protocols (Laver et al. 2015; Makuch and Johnson 1989; Saposnik and Levin 2011). We computed a NI value for two time intervals: postassessment and long-term follow-up. For example, for a patient who followed RGS training with a baseline, end of the treatment, and follow-up assessment, we calculated the NI per week for two periods: baseline to end of the treatment and end of the treatment to long-term follow-up. The measured change from baseline to the end of the treatment was allocated to the RGS or the OT group, while the change from the end of the treatment to follow-up was assigned to the follow-up group. If a patient had multiple follow-up assessments at different time points, all the follow-up measures were assigned to the follow-up group.

For all tests, statistical significance levels were set at *P* < 0.05. Average and dispersion values are reported in medians ± median absolute deviation (MAD) or means ± SD according to results from normality assessments (Kolmogorov-Smirnov normality test). To facilitate the exploration and replication of our findings, we published the complete data set and analysis source code as freely available and open, accessible at https://doi.org/10.5281/zenodo.3246368.

## RESULTS

### 

#### Impact assessment of individual conditions.

**Fig. 1.**
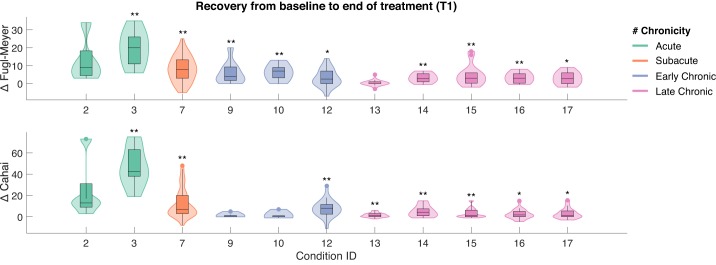
Effect of Rehabilitation Gaming System (RGS)-based treatment from the start (baseline) to the end of the treatment (T1). Impact measured on upper limb motor function in the Upper Extremity section of the Fugl-Meyer scale (UE-FM; *top*) and performance in Instrumental Activities of Daily Living (iADLs) captured by the Chedoke Arm and Hand Activity Inventory (CAHAI; *bottom*). The effect represents a change in each scale from the start to the end of the treatment. Notice that the horizontal axis refers to the RGS conditions listed in Table 1 and follows the same order. Shaded areas indicate the data distribution color coded according to the chronicity of stroke patients participating in each rehabilitation condition: acute (green), subacute (orange), early (blue), and late (purple) chronic stage. *P < 0.05, **P < 0.01.

The complete data set includes 219 stroke survivors assigned to 17 rehabilitation conditions (Table 1) at different stages post-stroke: acute (<3 wk), subacute (3 wk–6 mo), early chronic (6–18 mo), and late chronic (>18 mo). We observed significant gains in body function and structure after treatment, both in acute (median 20.0 ± 7.9 MAD, *P* < 0.01) and subacute patients (median 8.0 ± 5.6 MAD, *P* < 0.01), as measured by UE-FM (Fig. 1*A*; Table S1 in Supplemental Material). These gains were accompanied by an improved performance in iADLs in both acute (median 42.5 ± 14.1 MAD, *P* < 0.01) and subacute groups (median 7.0 ± 10.5 MAD, *P* < 0.01) (Fig. 1*B*; Table S2 in Supplemental Material). More interestingly, at the chronic and late chronic stage, the therapy showed overall effectiveness in facilitating improvements in UE-FM (ranging from median 2.7 ± 3.8 MAD to median 7.0 ± 3.6 MAD, *P* < 0.05) and CAHAI (median 1.0 ± 3.8 MAD to median 8.0 ± 5.6 MAD, *P* < 0.05). The application of the RGS at home showed no significant effects in UE-FM but induced statistically significant gains in the execution of iADLs (median 1.0 ± 1.6 MAD, *P* < 0.01). Surprisingly, a dosage-matched RGS study conducted in the clinic on late chronic patients had an impact on UE-FM (*condition 15* in Table 1, median 3.0 ± 4.1 MAD, *P* < 0.01; Fig. 1, *A* and *B*, condition Late Chronic 3w). Furthermore, we observed a clear dependency between the number of days post-stroke before the start of the RGS therapy and the improvements in motor function as measured by UE-FM and CAHAI (*P* < 0.001, Spearman correlation).

The analysis of follow-up measures illustrates that improvements were retained in all groups. The subacute group training with RGS exhibited a significant improvement during the follow-up period (3 mo after the end of treatment) both in UE-FM (median 2.0 ± 5.3 MAD, *P* < 0.01) and CAHAI (median 3.0 ± 11.7 MAD, *P* < 0.01) (Fig. S3, *A* and *B*, in Supplemental Material). The acute groups, however, showed higher interindividual variability and nonsignificant gains from the end of the therapy to the follow-up.

#### Revealing an extended critical window for recovery.

**Fig. 2.**
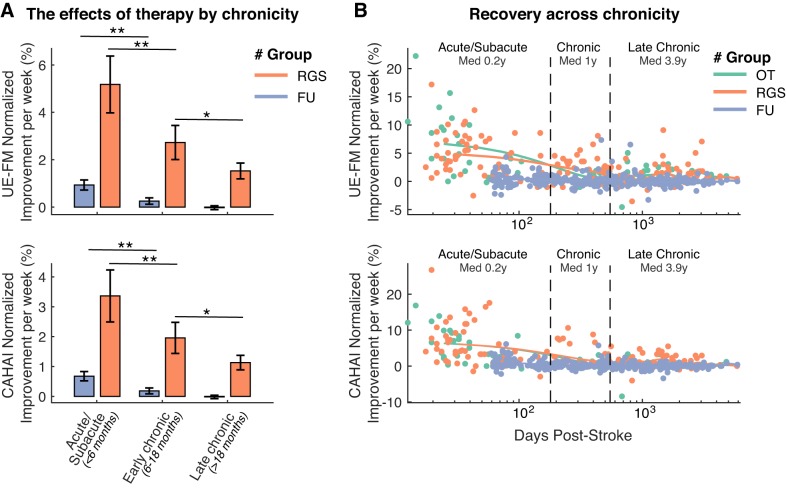
*A*: averaged normalized improvement rates per week after the Rehabilitation Gaming System (RGS)-based treatment and at follow-up (FU) for Upper Extremity section of the Fugl-Meyer scale (UE-FM; *top*) and Chedoke Arm and Hand Activity Inventory (CAHAI) scales (*bottom*) by patient’s chronicity at the time of the evaluation. The number of observations is indicated within or above each bar. *P < 0.05, **P < 0.01. *B*: comparison of the RGS, occupational therapy (OT), and FU measures of normalized improvement rates per week for UE-FM (*top*) and CAHAI (*bottom*) scales, by patient’s chronicity at the time of the evaluation. Solid lines indicate the average estimates based on the Whittaker smoothing algorithm (Eilers 2003). Vertical dashed lines indicate the limits of the 3 chronicity categories.

To study the temporal structure of recovery after a stroke, we now combined the impact of all the conditions and examined the effects of chronicity on the patients’ normalized improvement (NI; see *Eq. 1* in materials and methods). In the group receiving therapy (RGS), we observed a mean UE-FM NI per week of 5.2 ± 1.0 SD % in subacute (median 10.3 wk) and 2.7 ± 0.6 and 1.4 ± 0.3 SD % in early chronic (median 12.0 mo) and late chronic (median 3.9 yr) patients, respectively (Fig. 2, *top left*). The change on the CAHAI scale shows a mean NI per week of 3.4 ± 0.7 SD % in subacute, and 1.9 ± 0.4 and 1.1 ± 0.2 SD % in early and late chronic patients, respectively (Fig. 2, *bottom left*). We found statistically significant differences between subacute and early chronic patients, even during the follow-up period (*P* < 0.01, Wilcoxon Rank-Sum). Those patients at the subacute phase showed higher NI per week. However, only in the RGS group, the early chronic group (6–18 mo) showed higher recovery rates than the late chronic group (>18 mo) (*P* < 0.05, Wilcoxon Rank-Sum). This analysis reveals a long-lasting gradient of sensitivity to treatment that remains visible across the first 18 mo post-stroke (Fig. 2, *right*). This effect was not present in the follow-up measures, a period in which no therapy was administered. Due to the low number of chronic patients in the OT group, we could not apply the bootstrapping method to this sample for comparison. Despite this, we display the full data to allow for visual inspection.

The patient’s age could not explain the gradient of sensitivity to treatment found in the RGS group even at early and late chronic stages (Spearman’s correlation *r* < 0.003, *P* > 0.96) and neither by the patient’s baseline impairment score (Spearman’s correlation *r* < 0.052, *P* > 0.43 for FM; *r* < 0.006, *P* > 0.93 for CAHAI). Notice that the design of this analysis controls for additional confounding variables since all patients were recruited according to standard inclusion and exclusion criteria concerning age, motor impairment severity, cognitive impairment severity, type of stroke (Oxford Classification), hand dominance, the absence of a second stroke, and gender. None of these variables correlate with the patients’ chronicity, and therefore none of them can explain the uncovered gradient. The homogeneity of the demographics of these patients in combination with highly heterogeneous chronicity highlights the peculiarity of this data sample.

**Fig. 3.**
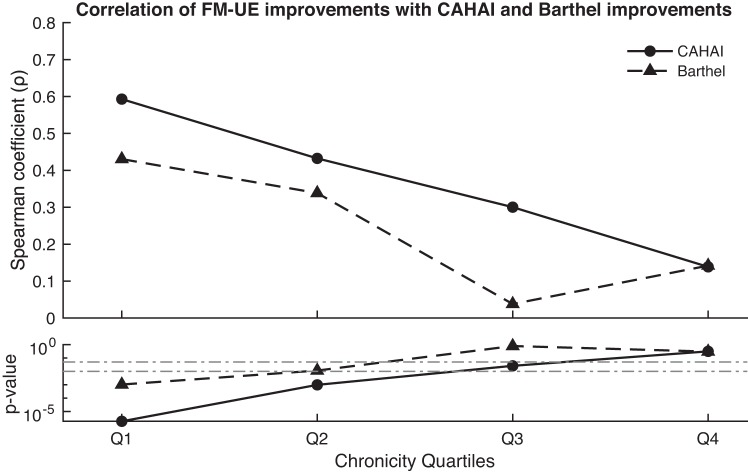
Spearman correlation coefficients of recovery scores captured by the Upper Extremity section of the Fugl-Meyer scale (UE-FM), Chedoke Arm and Hand Activity Inventory (CAHAI), and Barthel Index (BI), for each chronicity quartile. Q1 (2–39 days), Q2 (39–430 days), Q3 (439–1,198 days), and Q4 (1,198–5,844 days). Dashed horizontal lines indicate significance thresholds.

Finally, we analyzed the covariation of the recovery measures of the different clinical scales. We observed a distinct effect of the patient’s chronicity on the association of ∆UE-FM, ∆CAHAI, and ∆BI scores. While at the acute/subacute stages these recovery measures correlate, they do progressively dissociate as the patient advances toward the late chronic stage (Fig. 3).

## DISCUSSION

In opposition to compensation, recovery of body function and structure (Levin et al. 2009) [i.e., “true recovery” according to (Bernhardt et al. 2017)] refers to the partial or complete restoration of the repertoire of behaviors that was available before injury (Bernhardt et al. 2017; Zeiler and Krakauer 2013). In the absence of a precise assessment of kinematic and kinetic measures, recovery of body function and structure has been operationalized as the change in UE-FM scores (Kwakkel et al. 2017). Previous studies on humans have identified a 3- to 6-mo period of enhanced neuroplasticity mechanisms triggered by the injury (Hendricks et al. 2002; Kitago and Krakauer 2013). Here, by analyzing clinical recovery scores from stroke patients with variable chronicity but comparable baseline impairment levels, we are able to detect a smooth decrease in the sensitivity to treatment (i.e., critical window for recovery) that extends beyond 12 mo post-stroke. These results suggest that there is a long-lasting critical period of enhanced neuroplasticity post-stroke that enables improvement in body function and structure even at late chronic stages. This is the first time that such an extended critical period of recovery is reported. Capturing this effect may require large homogeneous data sets and analytic methods with enhanced accuracy such as the bootstrapping technique we apply here.

In line with the previous literature, our data illustrate the correlation of ∆UE-FM with ∆CAHAI and ∆BI scores at acute stages (Beebe and Lang 2009; Rabadi and Rabadi 2006). However, we found that at chronic stages these scales dissociate, possibly due to the introduction of compensatory mechanisms (Rabadi and Rabadi 2006). Altogether, our data support the interpretation of UE-FM as an assessment of recovery of body function and structure that is independent of improvement in the performance of iADLs and closely associated with true neurological repair (Kwakkel et al. 2004).

The results presented in this study do require further investigation for a number of reasons. The clinical importance of the detected improvements is marginal at late chronic stages. It is relevant to notice, however, that the UE-FM minimal clinically important difference (MCID) is derived from the chronicity-dependent variability in the UE-FM’s measurement error and the patient’s perceived improvement thresholds (Page et al. 2012). According to previous studies, the UE-FM MCID ranges from 16 to 30% of the scale’s maximum value at acute stages (<30 days post-stroke) (Lang et al. 2008), and from 7.2 to 11.0% at chronic stages (>4 mo post-stroke) (Page et al. 2012). In the case of CAHAI, MCID thresholds have been established above 7% of the scale range (6.3 points) for “stable patients” within the first year post-stroke (Barreca et al. 2005). If the reduction of the sensitivity to treatment revealed by our analysis exists and extends beyond 12 mo post-stroke, these MCID estimates could be better described as a continuous function of chronicity and should differentiate between late chronic and early chronic patients. Future studies should explore this relationship to delineate more accurate MCID thresholds.

It is important to note that the uncovered gradient of sensitivity to therapy may not be specific to VR-based interventions. However, due to the low number of chronic patients in the OT group, we could not perform a bootstrapping analysis in this sample, and we were thus not able to evaluate the limits of the critical window in these patients. Therefore, the generalization of our findings to therapies based on different rehabilitation methods (e.g., Constraint-Induced Movement Therapy) needs to be investigated. The factors determining the duration of this critical window and the decay rate of the patient’s responsiveness to treatment deserve further investigation.

Our results suggest that, as during ontogenesis, the reacquisition of function after stroke might have to be seen as a process that must satisfy distinct dependencies. Multiple sensitive periods for the acquisition of motor and cognitive function may be structured according to specific dependencies. For instance, in postnatal stages, the development of neural pathways for sensory processing precedes those of language and motor functioning (Hensch 2005). Based on our results, we speculate that the temporal structure of recovery post-stroke may also comprise such a cascade of domain-specific stages or critical periods. Clinical protocols for rehabilitation should be evaluated in this new context.

### 

#### Conclusion.

We have investigated the distinct dynamics of post-stroke recovery and the sensitivity to treatment. By unifying results from 11 rehabilitation pilot studies, we observed improvement in function over at all stages post-stroke. This effect displayed a specific gradient of recovery that faded out exponentially and reached asymptotic levels after one year and a half post-stroke. These findings call for an urgent scientific effort to reassess the critical window for recovery and highlight the need for both providing early therapy and extending it to patients in the chronic stages post-stroke.

## GRANTS

This study was supported by the Rehabilitation Gaming System, AAL Joint Program 2008-1, the European Commission (EC), the European Research Council under grant agreement 341196 (CDAC), EC H2020 project socSMCs (H2020EU.1.2.2. 641321), MINECO project SANAR (Gobierno de España) under agreement TIN201344200REC, and by the EIT Health project 19277. EIT Health is supported by EIT, a body of the European Union.

## DISCLOSURES

P. F. M. J. Verschure leads the research group SPECS that developed RGS, and is the CEO/founder of the spin-off company Eodyne Systems, SL, which commercializes RGS with the goal to achieve a large-scale distribution of low-cost science-based rehabilitation technologies.

## AUTHOR CONTRIBUTIONS

B.R.B., M.M., A.D., M.C., S.B., E.D., A.C., S.R., R.M.S.S.M., and P.F.V conceived and designed research; B.R.B., M.M., M.C., and S.B. performed experiments; B.R.B. and A.D. analyzed data; B.R.B., E.D., A.C., S.R., R.M.S.S.M., and P.F.V. interpreted results of experiments; B.R.B. prepared figures; B.R.B. and P.F.V. drafted manuscript; B.R.B., M.M., E.D., A.C., S.R., R.M.S.S.M., and P.F.V. edited and revised manuscript; B.R.B., M.M., A.D., M.C., S.B., E.D., A.C., S.R., R.M.S.S.M., and P.F.V. approved final version of manuscript.

## ENDNOTE

At the request of the authors, readers are herein alerted to the fact that additional materials related to this manuscript may be found at https://doi.org/10.5281/zenodo.2611949. The “Supplementary Material” within the file inventory has been peer reviewed. The remainder of these materials are not a part of this manuscript and have not undergone peer review by the American Physiological Society (APS). APS and the journal editors take no responsibility for these materials, for the Web site address, or for any links to or from it.

Data set D1, “PatientsDemographicsAndClinicalScreening.xlxl,” contains demographical data and clinical screening information (age, gender, chronicity, center ID, stroke type, Oxford classification, affected arm, arm dominance, presence of aphasia, days after stroke). Data set D2, “ClinicalScalesAll.csv,” contains recovery scores from 219 hemiparetic stroke patients evaluated using the Upper Extremity section of the Fugl-Meyer, CAHAI, and BI clinical scales at multiple time points (baseline, end of the treatment, and follow-up periods).
